# Genomics in the Czech Republic

**DOI:** 10.1002/cfg.327

**Published:** 2003-10

**Authors:** Václav Paçes

**Affiliations:** Institute of Molecular Genetics Academy of Sciences Flemingovo 2 Prague CZ-16637 Czech Republic


**Comparative and Functional Genomics**
*Comp Funct Genom* 2003; **4**: 518–519.
Published online in Wiley InterScience (www.interscience.wiley.com). **DOI:** 10.1002/cfg.327

**Conference Commentary**

### Genomics in the Czech Republic

Václav Paçes*
*Institute of Molecular Genetics, Academy of Sciences, Flemingovo 2, CZ-16637 Prague, Czech Republic*

**Correspondence to:
Václav Paçes, Institute of Molecular Genetics, Academy of Sciences, Flemingovo 2, CZ-16637 Prague, Czech Republic.
E-mail: vpaces@img.cas.cz*

Received: 1 August 2003
Revised: 4 August 2003
Accepted: 5 August 2003

The Functional Genomics and Disease 2003 conference was held at the Hilton Hotel in Prague, the beautiful capital of the Czech Republic, on 14–17 May 2003. This conference provided an ideal forum for European specialists to converge in the geographical centre of Europe to discuss the progress made in, and future prospects for, the important field of genomics in disease, and presented an opportunity to forge new collaborations. It was both an honour and a pleasure for Czech biochemists and biologists to organize and host the conference.

I would like to take this opportunity to inform you where research in genomics currently stands in the Czech Republic. Science in the Czech Republic is supported by several sources. Most of our support comes from the state budget and is distributed by several ministries, by the Academy of Sciences, and by the independent grant agency. Most of these bodies allocate money for research in genomics. In addition, a new structure was launched in 2002, the so-called 'Research Centres'. One of them is the Centre for Integrated Genomics. It consists of carefully selected relevant laboratories from the Institute of Molecular Genetics and Institute of Physiology (both from the Academy of Sciences of the Czech Republic), laboratories of the Institute of Inborn Metabolic Disorders of the Charles University Medical School, and several laboratories of the Institute of Chemical Technology. The Centre is supported by the Ministry of Education, Youth and Sports. Altogether 40 scientists, most of them under 30 years of age, focus their efforts on developing genomics methodologies, sharing them with other scientists and using them in several scientific projects. At present these projects range from bacterial comparative genomics to searching for retroviral elements in the human genome and identification of specific genes in various organisms. More practically orientated projects are aimed at genomics of inborn errors and environmental genomics (using bacterial genes for bioremediation).

There are other research centres that are using a genomics approach in a variety of projects, namely the Centre for Molecular and Cellular Immunology and the Centre for Molecular and Gene Biotechnology. The genomics network thus created now makes it possible to develop new projects at a state-of-the-art level.

One should realize that molecular genetics, genomics, proteomics and related fields started when the Czech Republic was still politically isolated from the democratic world. This also led to scientific isolation because it was impossible to collaborate freely. We hope that we have overcome this and that we have succeeded in catching up with the new trends in science. This has been facilitated

Copyright © 2003 John Wiley & Sons, Ltd.

by organizations such as the European Molecular Biology Organization (EMBO), the European Science Foundation (ESF) and the Federation of European Biochemical Societies (FEBS). It is important that our best laboratories are included in the 6th Framework Program of the European Union. Next year the Czech Republic will join the EU, and this will complete our full integration. I hope that the conference has contributed to this trend, that new collaborations and friendships will have started there, and that similar conferences will follow. Czech scientists are ready to help.

Copyright © 2003 John Wiley & Sons, Ltd.

